# Prognostic model for time to achieve independent walking in children with Guillain-Barré syndrome

**DOI:** 10.1038/s41390-021-01919-3

**Published:** 2022-02-15

**Authors:** Peerada Chaweekulrat, Oranee Sanmaneechai

**Affiliations:** 1 Department of Pediatrics, Faculty of Medicine Siriraj Hospital, Mahidol University, Bangkok, Thailand; 2 Center of Excellence for Neuromuscular Diseases, Faculty of Medicine Siriraj Hospital, Mahidol University, Bangkok, Thailand

## Abstract

**Background:**

Guillain-Barré Syndrome (GBS) is an immune-mediated peripheral neuropathy. Clinical features and outcomes in children differ from adults. Currently, there is no prognostic model to predict outcomes in children and existing models for adults are not suitable.

**Objectives:**

To identify factors that are associated with outcomes and develop clinical model to predict time to independent walking in children with GBS.

**Methods:**

Between 2005 and 2018, 41 patients with GBS were identified by retrospective chart review. Factors associated with independent walking were analyzed with the Kaplan–Meier method. A prediction model was developed based on regression coefficients from Cox’s proportional hazard model.

**Results:**

The disability score at maximum weakness and nerve conduction study results were associated with independent walking and included in the model. Scores range from 0 to 5. A score of 5 predicts 34 days to independent walking while a score of 0 predicts 5 months (mean 158 days, *p* = 0.008).

**Conclusion:**

This scoring system for pediatric patients provides predicts the time needed to achieve independent walking, an important milestone of recovery for communication with parents, and to assist clinicians to optimize treatment. Further studies of predictive factors and external validation are needed to improve precision of the model.

**Impact:**

- This is the first study to create a prognostic scoring system for individual outcomes in children with GBS.
- A clinical prognostic model can predict time to achieve independent walking in individual pediatric patients with GBS.
- This model can assist clinicians to optimize treatment and guide decisions on rehabilitation to prevent long-term disability.

## Introduction

Guillain-Barré Syndrome (GBS) is a severe, acute, post-infectious, immune-mediated peripheral neuropathy. The annual incidence of GBS in children under 15 years old ranges from 0.34 to 1.34 per 100,000.^1,2^ Patients usually present with progressive monophasic ascending muscle weakness, sensory deficit, and diminished deep tendon reflexes.^1,2^ Clinical features and outcomes in children differ from adults. The most common presenting symptoms of GBS in children are difficulty walking and pain.^3,4^ The disease varies from mild to severe, including the inability to walk independently and respiratory failure. Mortality occurs in fewer than 2% of children with GBS.^3^ The most common causes of death are pulmonary complications or autonomic dysfunction, including cardiac arrhythmias.^1^

Compared to adults, the prognosis in children is favorable.^5,6^ Ninety percent of pediatric patients will be able to walk independently within 6 months^3^ and 97.6% will be able to walk independently within 1 year after the onset of weakness,^7^ compared to only 80% in adult patients at 6 months^1,5^ and 84% at 1 year.^8^ In adults, poor outcomes are associated with age more than 40 years, preceding diarrhea, and a high GBS disability score.^9^ The Erasmus GBS outcome score (EGOS) is a clinical model that helps clinicians predict the probability of adult GBS patients being unable to walk independently during the first 6 months of follow-up.^9,10^ In children, poor outcomes are associated with age ≤5 years, preceding diarrhea, a disability score >3, autonomic dysfunction and cranial nerve (CN) involvement, and the absence of compound muscle action potential.^7,11–15^

There is no published clinical model that enables prediction of outcomes in children with GBS. Therefore, we aimed to identify factors that are associated with outcomes, and to develop a clinical model to predict time to independent walking in children with GBS.

## Methods

### Study design and study population

We performed a single-center retrospective chart review at Siriraj hospital, Mahidol University, Bangkok, Thailand during 2005–2018. The diagnosis of GBS was made according to the Asbury and Cornblath criteria, and patients <18 years old who were admitted to the hospital or managed in the outpatient department were included.^16^ Patients that were lost to follow-up after discharge were excluded. Age, sex, history of preceding infection, clinical features (weakness, pain, reflex, autonomic dysfunction, CN involvement and ataxia), duration of mechanical ventilation, cerebrospinal fluid (CSF) analysis, and electrophysical findings were collected. All patients were followed during outpatient visits and the GBS disability score was recorded until the final follow-up visit. The GBS disability score was defined according to criteria established by Hughes et al.^17^. A disability score of 0 represents a normal condition; 1 indicates the patient has mild symptoms and is capable of running; 2 indicates the patient is able to walk 10 meters independently but is unable to run; 3 indicates the patient is able to walk only with assistance; 4 indicates the patient is bedridden or chair-bound; 5 indicates the patient is mechanically ventilated, and a score of 6 indicates the patient is deceased. A good outcome for GBS patients is defined as the ability to walk independently (i.e., GBS disability score of 0, 1, 2). The time in days from symptom onset to outcome was recorded. According to an electrophysical analysis based on the criteria introduced by Cornblath, patients were classified into either acute inflammatory demyelinating polyneuropathy (AIDP), acute motor axonal neuropathy (AMAN), acute motor sensory axonal neuropathy (AMSAN), or Miller-Fisher syndrome (MFS).^18^ Patients without electrophysical data from a nerve conduction velocity study were considered to be an unclassified subtype. The study was approved by the Siriraj Institutional Review Board COA no. 511/2562(EC3).

### Data analysis

Demographic data were presented using descriptive statistics. Univariate analysis of factors associated with time to independent walking was performed using the Kaplan–Meier method. Factors that had a *p* value of <0.05 in the univariate analysis were entered into the Cox’s proportional hazard (PH) model. The regression coefficients (*b*) were used to create scores. The coefficient from each predictor was initially divided by the smallest coefficient and then rounded up to simplify the score calculation. Statistical analyses were performed using PASW 18.0. A *P* value of <0.05 was considered to be significant.

## Results

### Clinical characteristics

There were 52 children with a diagnosis of GBS and 41 patients were included in this study. Six patients received care at other hospitals and five patients were lost to follow-up after discharge. The patient’s ages ranged from 3 months to 17.9 years with a median of 6.3 years. Thirty patients (73%) were male and the male to female ratio was 2.7:1. Baseline clinical characteristic are summarized in Table 1. Twenty-five patients (61%) reported an infection 1–4 weeks before the onset of weakness. No patient had a history of vaccination 1–4 weeks prior to the onset of weakness. All patients presented with ascending symmetrical weakness and hyporeflexia or areflexia at the time of diagnosis. Sensory impairment was found in 13 of 29 patients (48.8%) that cooperated with a sensory examination. CN involvement was present in 12 patients (5 with ophthalmoplegia, 6 with facial palsy, and 1 with an absent gag reflex). Autonomic dysfunction was present during the first week of admission in eight patients (7 hypertension, 1 urinary retention). Ataxia was present in two of 30 examined patients (6.7%). Ten patients (24.4%) were mechanically ventilated during hospitalization with a mean duration of 13.6 days (range 3–31 days). 77.5% of patients had cytoalbumino-dissociation in the CSF. A nerve conduction study (NCS) was done in 30 patients (73.2%). NCS revealed demyelination in 14 patients and an axonal subtype in 16 patients. The GBS subtypes are described in Table 1. Eleven patients without NCS were considered to be an unclassified subtype.

**Table 1:** Baseline clinical characteristics of children with Guillain-Barré Syndrome (*n* = 41).

Characteristic	No. (%)
Age (years)^a^	6.3 (0.33–17.9)
**Male**	30 (73)
Antecedent infection	
Upper respiratory tract infection	16 (39)
Gastrointestinal infection	5 (12.2)
Other	4 (9.8)
GBS subtype	
AIDP	14 (34.1)
AMAN	13 (31.7)
AMSAN	2 (4.9)
MFS	1 (2.4)
Unclassified	11 (26.8)
Clinical presentation	
Weakness	41 (100)
Sensory impairment (*N* = 29)	13 (44.8)
Cranial nerve involvement	12 (29.2)
Autonomic dysfunction	8 (19.5)
Ataxia (*N* = 30)	2 (6.7)
CSF protein (mg/dl)^a^	86.5 (12–529)
CSF protein > 40 mg/dl	31 (77.5)

^a^Data presented as median (range).

Intravenous immunoglobulin 2 g/kg was given to 35 patients (85.4%). The mean time to receive treatment was 7 days after the onset of weakness. Six patients who could ambulate without assistance did not receive immunoglobulin treatment. The median time to maximum weakness was 10 days after the onset of weakness. Disease severity at maximum weakness and follow-up time are shown in Table 2.

**Table 2:** Severity of GBS at maximum of weakness and at follow-up visits (*n* = 41).

GBS disability score		At maximum of weakness^a^ (*n* = 41)	Outcome at 60 days (*n* = 41)	Outcome at 180 days (*n* = 30)	Outcome at 1 year (*n* = 23)
0	Healthy state	0 (0)	0 (0)	1 (3.3)	4 (17.4)
1	Minor symptoms with capable running	0 (0)	12 (29.3)	15 (50.0)	13 (56.5)
2	Walks without assistance for 10 meters but unable to run	6 (14.6)	20 (48.8)	10 (33.3)	4 (17.4)
3	Able to walk with assistance	8 (19.5)	4 (9.8)	3 (10)	1 (4.3)
4	Bedridden or chairbound	17 (41.5)	6 (14.6)	1 (3.3)	1 (4.3)
5	Mechanically ventilated	10 (24.3)	0 (0)	0	0

^a^Median days to maximum of weakness (min–max) = 7 days (1–54).

### Outcome

The median follow-up time was 264 days (range 26–2305). No patient died and GBS reoccurred in one patient (Table 2). The median time to achieve independent walking was 38 days (range 5–331). Thirty-nine (95%) patients were able to walk independently at their final follow-up visit.

### Factors associated with time to achieve independent walking in GBS patients

**Fig. 1:**
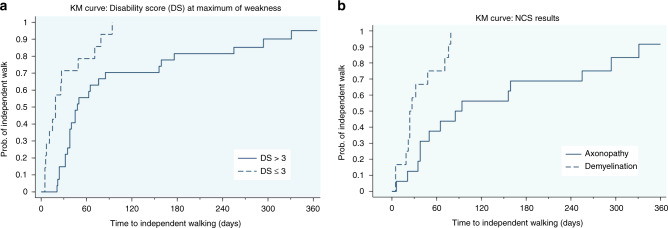
Kaplan–Meier curve of probability to achieve independent walking (0–360 days). **a** Disability score: Difference between disability score of ≤3 and >3 (*P* = 0.002). Solid line represents probability of independent walking in disability score >3 group and dashed line represents probability of independent walking in disability score ≤3 group. **b** NCS results: Difference between Demyelination and Axonopathy (*P* = 0.001). Solid line represents probability of independent walking in axonopathy group and dashed line represents probability of independent walking in demyelinating group.

Univariate analysis identified several factors that were associated with independent walking including female, age < 6 years, disability score ≤ 3, no proceeding GI illness, presence of CN involvement, no respiratory failure, and demyelination (Table 3). Only the presence of demyelination and a disability score ≤3 were significantly associated with an earlier time to achieve independent walking (*p* ≤ 0.05). Only demyelinationwas significantly associated with independent walking in the multivariate analysis (Table 4, Fig. 1). The probability of independent walking is provided in Table 5. All (14) patients with evidence of demyelination from the NCS walked independently within three months, but two (8%) of patients with axonopathy were still unable to walk independently 1 year after symptom onset.

**Table 3:** Factors affecting time to independent walking.

Factor		*n*	Independent walking *n* (%)	Median days to independent walking	Crude HR (95% CI)	*P* value
Sex	Male	30	28 (93.3)	45	1	
	Female	11	11 (100)	38	1.51 (0.74–3.09)	0.259
Age	<6	18	18 (100)	38	1.40 (0.73–2.69)	0.306
	≥6	23	21 (91.3)	48	1	
Highest disability score	≤3	14	14 (100)	19	2.91 (1.48–5.86)	0.003
	>3	27	25 (92.6)	48	1	
Preceding GI symptoms	Absence	36	35 (97.2)	40	1.52 (0.53–4.32)	0.432
	Presence	5	4 (80)	38	1	
Cranial nerve involvement	Absence	27	26 (96.3)	45	1	
	Presence	12	12 (100)	32	1.57 (0.78–3.17)	0.205
Sensory involvement	Absence	16	15 (93.7)	35	1.07 (0.48–2.34)	0.876
	Presence	13	12 (92.3)	38	1	
Autonomic dysfunction	Presence	28	28 (100)	38	0.88 (0.39–19.72)	0.749
	Absence	8	8 (100)	45	1	
Reflex	Areflexia	31	30 (96.8)	45	1	
	Hyporeflexia	9	8 (88.9)	38	0.81 (0.37–1.77)	0.589
Respiratory failure	Absence	31	30 (96.8)	38	1.61 (0.76–3.43)	0.217
	Presence	10	9 (90)	63	1	
Cytoalbumino-dissociation	Absence	10	9 (90)	45	0.65 (0.31–1.39)	0.268
	Presence	30	29 (96.7)	38	1	
NCS results	Demyelination	14	14 (100)	24	4.37 (1.68–11.39)	0.003
	Axonopathy	16	14 (87.5)	85	1

**Table 4:** Multivariate Cox’s regression analysis of factors associated with time to independent walking.

Factor		b^a^	b/smallest b	Adjusted HR (95% CI)	*P* value
Disability score	≤3	0.35	1	1.42 (0.52–3.85)	0.493
	>3			1	
NCS results	Demyelination	1.32	4	3.74 (1.29–10.82)	0.015
	Axonopathy			1	

^a^b = Regression coefficient.

**Table 5:** Prediction of time to independent walking.

Factors	Time to independent walking^a^	% Able to walk independently				
		1 months	2 months	3 months	6 months	12 months
NCS result						
Demyelination	24 (2, 18.3–29.7)	58	75	100	100	100
Axonopathy	85 (29, 28.2–141.8)	13	38	50	69	92
Highest disability score						
≤3	19 (20, 15.2–46.7)	71	79	93	100	100
>3	48 (8, 35.3–60.7)	15	56	70	81	95

^a^Presented as median (SE, 95% CI).

### Clinical prediction model to predict time to independent walking

**Fig. 2:**
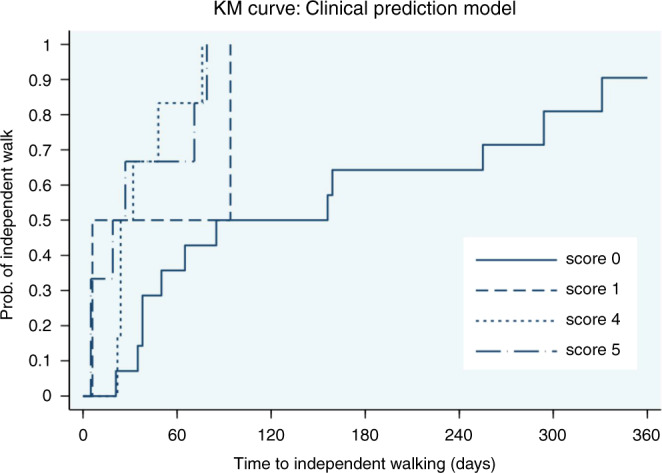
Kaplan–Meier curve of probability to achieve independently walking in each score group (0–360 days). Scores range from 0 to 5 (*P* = 0.008). Solid line represents score 0. Dashed line represents score 1. Dotted line represents score 4 and dash dotted line represents score 5.

The disability score at the maximum of weakness and the NCS results were entered into were included in a model to predict time to independent walking in each patient. The Cox’s regression coefficients (*b*) were used to create the scoring system. The coefficient from each predictor was first divided by the smallest coefficient and then rounded up to simplify the score calculation. Predictive scores ranged from 0 to 5 (Table 6). The Kaplan–Meier curve (Fig. 2) displays the probability of achieving independent walking by time in each score group. Patients with a score of 5 required a mean of 34 days after the onset of weakness to achieve independent walking. Conversely, patients with score of zero required a mean of 158 days to achieve independent walking (*p* = 0.008).

**Table 6:** Predicted outcome by individual score.

Scores	Mean days to independent walking^a^	% Independent walking				
		1 months	2 months	3 months	6 months	12 months
0	158 (33.3, 92.9–223.6)	7	36	50	64	90
1	50 (44)	50	50	50	100	100
4	38 (8.6, 20.7–54.6)	50	83	100	100	100
5	34 (13.4, 8.2–60.5)	67	67	100	100	100

^a^Presented as mean (SE, 95% CI).

## Discussion

This retrospective cohort study included patients managed over a 14-year period and with up to 8 years of follow-up to identify factors associated with time to independent walking. These data enabled us to develop the first model that can predict the time needed for pediatric GBS patients to walk independently.

Our patients were predominantly male and the median age was 6 years, similar to a previous report.^3^ There were no deaths in our cohort while other studies have reported mortality of between 1 and 2%.^3,7,19^ One-fourth of our patients required mechanical ventilation, similar to the 10–30%^3,7,13,15,18–20^ rate reported in previous studies. The prevalence of GBS subtypes varies depending on the geographic region and the age of patients. The axonal subtype is more common in children than adults. Approximately 5–30% of adult patients are diagnosed with axonal subtypes,^21^ while 45–55% of children with GBS had the axonal subtype in previous reports.^3,7,13,22^ In our study, 34.1% of patients had the demyelinating subtype and 39% had the axonal subtype. This could be because our institution is a major tertiary referral center that receives more severe patients from around the country.

We found that 95.2% of pediatric GBS patients were able to walk independently within 1 year. Similarly, another study reported that 96% of patients recovered within 1 year with minimal functional deficit.^3^ We demonstrated that a disability score of ≤3 at the point of maximum weakness and the presence of demyelination on the NCS are associated with a reduced time needed to walk independently. Similarly, a high disability score has been correlated with poor outcomes.^7,12^ Evidence of demyelination from NCS is a powerful predictor of time to independent walking. However, NCS was not performed in all children because reliable electrophysical investigations are difficult to perform in pediatric patients because it is a painful procedure and patients are not always cooperative. Therefore, NCS is not conducted in most hospitals in Thailand.

The EGOS is a clinical prognostic scoring system for adult patients that is based on age >40 years, preceding diarrhea, and high disability score.^9,10^ In contrast, we developed a predictive scoring system using acute phase clinical features. Our model includes the GBS disability score at the point of maximum weakness and the results of nerve conduction studies. Patients with the lowest score had a probability of disability up to 10% at 1 year and needed much more time to be able to walk independently compared to patients with higher scores. However, the prognostic scoring system was derived from a retrospective data review in a distinct group of patients, which might limit its general applicability. In addition, the study was based in Thailand where the axonopathy type is common. Finally, despite a 14-year data collection period the statistical power of our study was reduced because the low prevalence of GBS limited our sample size.

This is the first study to create a prognostic scoring system for prediction of individual outcomes in children with GBS. It provides outcome as a function of time in days to achieve independent walking, an important milestone of recovery for communication with parents. The model can be used in the early phase of the disease and is suitable for countries where pediatric electrophysical investigation is limited. This scoring system can assist clinicians to optimize treatment for individuals and guide decisions on rehabilitation to prevent long-term disability. Further studies of predictive factors and external validation of the model with a larger dataset are planned to improve the accuracy of the scoring system.

## Supplementary information

Supplementary Information
