# Supplementary material for: Prognostic model for time to achieve independent walking in children with Guillain-Barré syndrome
Source: Pediatr Res. 2022 Feb 15;92(5):1417–22. doi: 10.1038/s41390-021-01919-3 (PMC9700508; doi:10.1038/s41390-021-01919-3)
Supplement: Supplementary file 1 — Supplementary Information [file 41390_2021_1919_MOESM1_ESM.pdf]

Figure 1. Kaplan-Meier curve of probability to achieve independent walking (0 – 360 days).

A. Disability score (P=0.002)

B. NCS results: Demyelination and Axonopathy (P=0.001)

A. B.

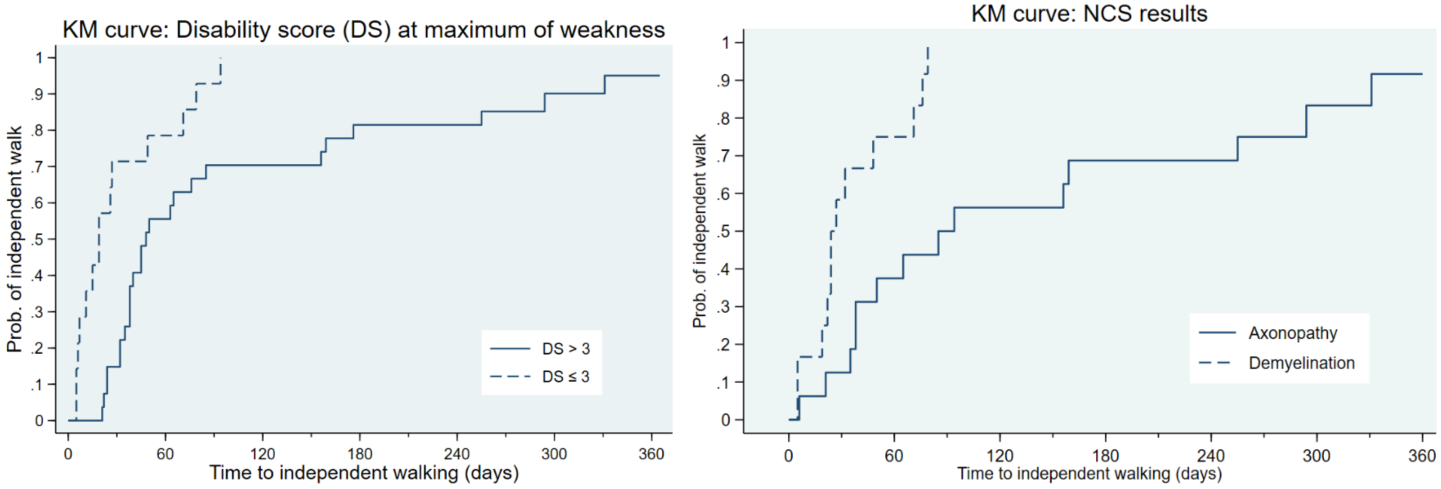

Figure 2. Kaplan-Meier curve of probability to achieve independent walking in each score group (0 – 360 days). (P=0.008)

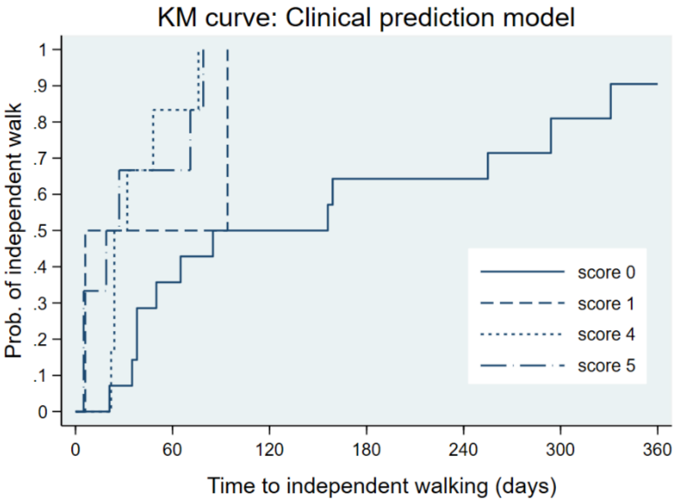

Factor	score
Disability score at maximum of weakness	
≤3	1
>3	0
NCS results	
Demyelination	4
Axonopathy	0
Score	0 – 5

GBS disability score [16]

0	Normal condition
1	Mild symptoms and capable of running
2	Able to walk 10 meters independently; unable to run
3	Able to walk only with assistance
4	Bedridden or chair-bound
5	Mechanically ventilated
6	Dead
